# Assessing the Prevalence of Ectopic Cerebellar Tonsils and Accompanying Symptoms in Individuals with Various Headaches

**DOI:** 10.5334/jbsr.3264

**Published:** 2023-10-03

**Authors:** Ghodsiyeh Azarkar, Sahar Nemati, Shabnam Vafadar, Gholam Reza Sharifzade, Arash Ziaee, Hossein Ashrafi

**Affiliations:** 1 Infectious Diseases Research Center, Birjand University of Medical Sciences, IR; 2 Department of audiology, razi Hospital, Birjand University of Medical Sciences, Birjand, IR; 3 Department of neurology, razi Hospital, Birjand University of Medical Sciences, Birjand, IR; 4 Department of Community Medicine, Faculty of Health, Birjand University of Medical Sciences, Birjand, IR; 5 Student Research Committee, Faculty of Medicine, Mashhad University of Medical Sciences, IR; 6 Department of Radiology, Valiasr Hospital, Birjand University of Medical Sciences, Birjand, IR

**Keywords:** Chiari malformation, cerebellar tonsil ectopia, headache, vertigo, nausea

## Abstract

**Objectives:**

Chiari malformation exhibits well-defined clinical signs, symptoms, and incidence rates in clinical studies. However, cerebellar tonsil ectopia presents with ambiguous symptoms and undetermined incidence rates in numerous studies. Our objective was to determine the incidence of cerebellar tonsil ectopia in individuals with headaches and identify additional clinical symptoms. This aims to improve diagnosis accuracy for clinicians and neurologists, leading to more effective treatment approaches.

**Methods:**

A cross-sectional study conducted in 2022 included 2305 participants aged 4–78 years presenting with headache. Chiari malformation was diagnosed using magnetic resonance imaging (MRI) scans, with a definition of cerebellar tonsil herniation extending beyond 5mm into the cervical canal.

**Results:**

The prevalence of Chiari malformation was 3.4%, with no significant gender difference (p = 0.72). There was a significant correlation between Chiari malformation and headache exacerbation due to the Valsalva maneuver (p < 0.001) and the presence of vertigo (p < 0.001). No significant association was found between Chiari malformation and accompanying symptoms such as nausea (p = 0.43), photophobia (p = 0.2), phonophobia (p = 0.52), and speech disorders (p = 0.45).

**Conclusion:**

These findings suggest a notable prevalence of Chiari malformation among headache patients and its association with specific headache characteristics, such as acute and occipital headaches, exacerbation by the Valsalva maneuver, and the co-occurrence of vertigo. These results underscore the need to consider Chiari malformation in the differential diagnosis of patients presenting with these specific headache features.

## Objectives

Chiari malformations type I (CMs) are classified as rare disorders, either congenital or acquired, marked by the unusual positioning of cerebellar tonsils beyond 5 mm below the spinal canal. This abnormal displacement can disturb the natural flow of cerebrospinal fluid, thereby causing symptoms such as headaches, syrinx, or hydrocephalus. Despite the rarity of this condition, it is significant to understand its implications due to its less than 1% prevalence rate in the United States [1,2].

Several congenital pathologies, including basilar invagination, platybasia, craniosynocytosis, posterior fossa hypoplasia, atresis of the foramina of Magendie and Luschka, enlarged cerebellar structures, and clival anomalies have been linked with CMs [3,4,5]. Additionally, congenital biomechanical deficiencies alongside secondary or iatrogenic factors such as trauma, hydrocephalus, tethered cord, and intraspinal hypotension have also been implicated in the etiology of this condition [6,7,8,9,10,11]. While the majority of CM cases remain asymptomatic in the initial stages, symptoms typically start manifesting between the ages of 25 to 40, ranging from mild headaches and dysesthesias to severe conditions such as respiratory failure and tetraplegia [3,4].

Interestingly, research has indicated a potential correlation between the length of herniated cerebellar tonsils and the severity of clinical manifestations [3,12,13,14]. Given the diverse extent of nervous tissue displacement, variations in the size of the foramen magnum, and the range of structures compressed, the clinical presentation of CMs is highly protean, posing a complex challenge for neurology practitioners. Increasing awareness about the different clinical presentations could foster more prompt recognition of the condition and timely magnetic resonance imaging (MRI) testing [3].

Headaches, arguably the most common manifestation, can sometimes be the sole indication of the presence of CMs or may be completely absent [15,16]. Characteristically, these headaches, associated with Chiari, are occipital-suboccipital in nature, often exacerbated by Valsalva maneuvers, positional changes, head dependency, and physical exertion [3,15].

Management strategies for CMs often revolve around decompressive suboccipital craniectomy, though the rationale behind the surgical intervention is a subject of ongoing debate [17]. Generally, conservative management without surgery is adopted for headaches unless the patient experiences significant disability due to a specific occipital-suboccipital headache accompanied by a concurrent cough headache.

This study aims to investigate the prevalence of Chiari malformations (CMs) and cerebellar ectopia in headache patients. By analyzing and discussing the accompanying symptoms, we aim to enhance understanding, facilitate timely diagnosis, and optimize management strategies for this complex neurological condition.

## Materials and Methods

### Study Design and Participant Recruitment

**Figure 1:**
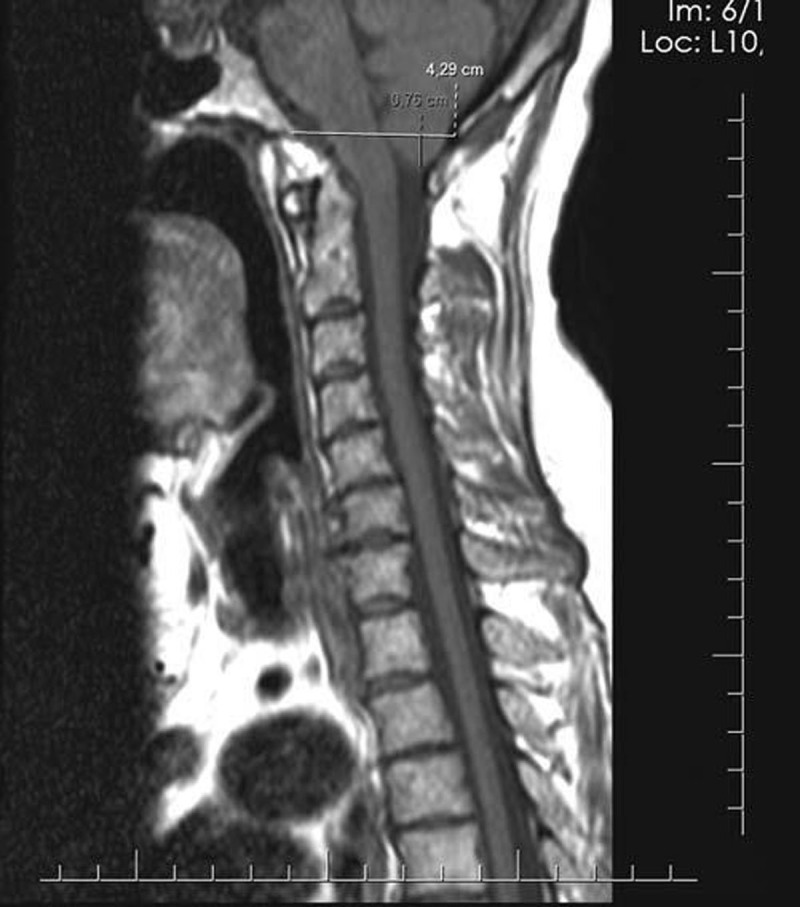
Sagittal T1-weighted TSE image showing cerebellar tonsils below the level of the foramen magnum.

We conducted a cross-sectional study in 2022 using a systematic random sampling method. The study participants were individuals of all ages who presented with headache symptoms and were referred to the MRI department. Exclusion criteria were applied to individuals with known brain disorders (such as tumors, multiple sclerosis, hydrocephalus), neurological symptoms (such as paresis, paresthesia), a history of head trauma, or prior brain surgery. The diagnosis of Chiari malformation was made based on cerebellar tonsil protrusion greater than 5 mm, and borderline diagnosis was determined by a descent of 3–5 mm into the foramen magnum, as observed in the midsagittal plane on the T1 sequence. Please refer to Figure 1 for a visual representation.

### Headache Definition and Classification

The classification of headaches was determined by two factors: acuteness and severity. The severity of headaches was defined as mild (a headache that somewhat interferes with the ability to function), moderate (a headache that hinders the ability to function but does not necessitate rest), and severe (a debilitating headache requiring rest in bed). Headache acuteness was classified as acute (lasting hours up to a day), subacute (lasting a day up to a month), and chronic (persisting for months or years).

## Results

### Participant Demographics and Chiari Malformation Prevalence

In a 2022 study at Vali-Asr and Imam Reza Birjand hospitals, 2305 individuals with headaches underwent MRI examinations. Among them, 79 patients (3.4%) showed cerebellar tonsil herniation exceeding 3 mm into the cervical canal. The prevalence of Chiari malformation and cerebellar tonsil ectopia based on herniation extent beneath the foramen magnum level is as follows:

Ectopia <3 mm: 1434 females (96.8%) and 792 males (96.2%).

Ectopia 3–5 mm: 27 females (1.8%) and 19 males (2.3%).

Ectopia >5 mm: 21 females (1.4%) and 12 males (1.5%).

Overall, 84.9% (1956 patients) had ectopia <3 mm, and 15.1% (349 patients) had ectopia ≥3 mm. Importantly, gender did not significantly influence the distribution of ectopia lengths (p = 0.72).

### Headache Intensity

A comparison of headache severity across healthy individuals, those with cerebellar tonsillar ectopia, and individuals diagnosed with Chiari malformation is provided in Table 1. The majority of patients from all three cohorts reported experiencing moderate headaches, with no substantial difference noted (p = 0.57).

**Table 1:** Comparison Of The Relative Frequency Of Cerebellar Tonsil Ectopia According To Severity.


	EXTENT	HEADACHE SEVERITY		

		MILD	MODERATE	SEVERE

Ectopia	<3 mm	172 (7.7%)	1859 (83.5%)	195 (8.8%)

	3–5 mm	4 (8.7%)	36 (78.3%)	6 (13.0%)

	>5 mm	3 (9.1%)	25 (75.8%)	5 (15.2%)

Total		179 (7.8%)	1920 (83.3%)	206 (8.9%)


### Headache Period

The study assessed the duration of headaches, noting varied results across different groups. Table 2 denotes the frequency of cerebellar tonsil ectopia corresponding to headache occurrence timing. Most Chiari malformation patients reported acute headaches (69.7%), while those with cerebellar tonsillar ectopia and the healthy group primarily reported subacute headaches (39% and 70%, respectively). The distinction between the Chiari malformation group and the cerebellar tonsillar ectopia group was statistically significant (p < 0.001).

**Table 2:** Comparison Of Ectopic Frequency Of Cerebellar Tonsils According To Time.


	EXTENT	HEADACHE PERIOD		

		ACUTE	SUBACUTE	CHRONIC

Ectopia	<3 mm	284 (12.8%)	1558 (70.0%)	384 (17.3%)

	3–5 mm	16 (34.8%)	18 (39.1%)	12 (26.1%)

	>5 mm	23 (69.7%)	5 (15.2%)	5 (15.2%)

Total		323 (14.0%)	1581 (68.6%)	401 (17.4%)


### Headache Localization

Headache localization varied significantly among the groups, as displayed in Table 3, which details the cerebellar tonsil ectopia frequency based on headache location. The majority of Chiari malformation patients experienced occipital headaches (87%), while cerebellar tonsil ectopia patients (39%) and healthy individuals (12%) reported headaches in diverse locations. This variation was statistically significant (p < 0.001).

**Table 3:** Comparison Of The Frequency Of Ectopia Of Cerebellar Tonsils According To Location Of Headache.


	EXTENT	LOCATION			

		FULL HEAD	OCCIPUT	FRONTAL	OTHER

Ectopia	<3 mm	1072 (48.2%)	274 (12.3%)	690 (31.0%)	190 (8.5%)

	3–5 mm	17 (37.0%)	18 (39.1%)	8 (17.4%)	3 (6.5%)

	>5 mm	2 (6.1%)	29 (87.9%)	1 (3.0%)	1 (3.0%)

Total		1091 (47.3%)	321 (13.9%)	699 (30.3%)	194 (8.4%)


### Valsalva Maneuver-Induced Headache Exacerbation

Table 4 examines the correlation between Chiari malformation, cerebellar tonsil ectopia and normal population and headache exacerbation triggered by the Valsalva maneuver. A significantly higher percentage of Chiari malformation (81.8%) and cerebellar tonsil ectopia patients (56.5%) reported positive Valsalva maneuver responses compared to healthy participants (13.3%) (p < 0.001).

**Table 4:** Comparison Of Frequency Of Ectopia Condition According To Exacerbation By Valsalva Maneuver.


	EXTENT	EXACERBATION BY VALSALVA MANEUVER	

		NEGATIVE	POSITIVE

Ectopia	<3 mm	1930 (86.7%)	296 (13.3%)

	3–5 mm	20 (43.5%)	26 (56.5%)

	>5 mm	6 (18.2%)	27 (81.8%)

Total		1956 (84.9%)	349 (15.1%)


### Accompanying Clinical Symptoms with Headache

Table 5 examined the occurrence of accompanying symptoms such as nausea, photophobia, phonophobia, and speech disorder in relation to headache, finding no significant differences among the three groups (p = 0.43, p = 0.2, p = 0.52, and p = 0.45, respectively). Furthermore, patients with Chiari malformation had a significantly higher incidence of vertigo (75.8%) compared to those with cerebellar tonsillar ectopia (17.4%) and healthy individuals (18%) (p < 0.001).

**Table 5:** Accompanying Clinical Symptoms with Headache.


ECTOPIA		NAUSEA	PHOTOPHOBIA	PHONOPHOBIA	SPEECH DISORDER	VERTIGO

<3 mm	Negative	1514 (68.0%)	1915 (86.0%)	1950 (87.6%)	2104 (94.5%)	1825 (82.0%)

	Positive	712 (32.0%)	311 (14.0%)	276 (12.4%)	122 (5.5%)	401(18.0%)

3–5 mm	Negative	32 (69.6%)	41 (89.1%)	41 (89.1%)	43 (93.5%)	38 (82.6%)

	Positive	14 (30.4%)	5 (10.9%)	5 (10.9%)	3 (6.5%)	8 (17.4%)

>5 mm	Negative	19 (57.6%)	25 (75.8%)	31 (93.9%)	30 (90.9%)	8 (24.2%)

	Positive	14 (42.4%)	324 (14.1%)	283 (12.3%)	3 (9.1%)	25 (75.8%)


## Discussion

Our comprehensive study conducted in 2022 involved a cohort of 2305 patients, with the primary objective of analyzing the prevalence, extent, and accompanying symptoms of Chiari malformation.

Our research, along with two other studies, investigated headache types and related symptoms in various patient groups. We found no significant differences in common symptoms among groups, implying these may not be key indicators for Chiari malformation or cerebellar tonsillar ectopia. However, a strong connection between cerebellar tonsil ectopia frequency and vertigo, particularly in Chiari patients, was discovered. This suggests the importance of considering non-traditional symptoms for diagnosis and treatment. Additionally, a study indicated a range of symptoms and an inverse relationship between tonsillar ectopia size and typical Chiari symptoms, implying a correlation between condition and symptom severity [18,19].

This study revealed that patients with <3 mm had higher pain scores than those in the 3–5 mm or >5 mm groups, suggesting varied headache severity in different groups. These results imply that conditions like Chiari malformation and cerebellar tonsillar ectopia can influence symptom severity, but no clear correlation was found with nausea and photophobia. There could, however, be a significant link between these conditions and symptoms like vertigo and certain headaches. More research is needed to improve symptom management understanding.

Our findings align with previous research [20,21,22] and demonstrate a significant association between Chiari malformation or cerebellar tonsil ectopia and exacerbated headaches during the Valsalva maneuver. Our results indicate a similar trend, with a higher proportion of patients experiencing increased headaches compared to healthy participants. Furthermore, our study reinforces the importance of distinguishing between headaches associated with Chiari malformation and primary cough headaches. These insights enhance our understanding of symptom management and contribute to more accurate differential diagnosis.

Our study revealed a strong association between the frequency of ectopia and the occurrence of vertigo. Chiari malformation patients had a significantly higher incidence of vertigo compared to those with cerebellar tonsillar ectopia and healthy individuals. Another study also found vertigo to be a common symptom in patients with symptomatic tonsillar ectopia, along with unstable gait and limb numbness. These findings indicate that neuroanatomical variations play a crucial role in the manifestation of vertigo. Consequently, there is potential for a targeted approach to diagnose and manage vertigo in patients with Chiari malformation and cerebellar tonsillar ectopia [23].

Our study, along with previous research, sheds light on the correlation between symptoms like nausea, photophobia, phonophobia, speech disorder, and headaches in three distinct groups categorized by the degree of ectopia. Consistent with earlier studies, we found no significant differences in these symptoms among the groups, indicating a universal occurrence rather than a connection with ectopia severity. However, when examining the association between ectopia frequency and vertigo incidence, our data aligns with previous research. Patients with Chiari malformation, characterized by greater ectopia, had a significantly higher incidence of vertigo compared to those with cerebellar tonsillar ectopia and healthy individuals. This emphasizes the importance of understanding the symptom profile in patients with varying degrees of ectopia, with vertigo being particularly linked to severity. Therefore, it is crucial to focus on effective symptom management, especially for vertigo in severe cases like Chiari malformation [24,25].

One notable limitation of this study lies in its cross-sectional design, which precludes establishing a cause-effect relationship between the observed symptoms and the presence of Chiari malformation or cerebellar tonsillar ectopia. The findings of this study are also contingent upon the reliability of participant self-reporting during interviews, which can be subject to recall bias or inaccurate reporting. Furthermore, the exclusion criteria applied may have potentially left out certain symptomatic cases with pre-existing brain disorders or prior brain surgeries.

## Conclusion

Our study found that symptoms were similar among patients with Chiari malformation and cerebellar tonsillar ectopia, except for a higher occurrence of vertigo in Chiari malformation cases. This highlights the need for a nuanced approach to diagnosis and management. Our results align with other studies, emphasizing diverse headache severity and the impact of physical manifestations on symptoms. We also discovered the significance of the Valsalva maneuver in triggering headaches, distinguishing them from benign cough-associated headaches. This correlation suggests a targeted approach to diagnosis and management. Overall, our findings stress the importance of understanding symptoms and effective management strategies for these conditions.

## Data Accessibility Statements

Prior to undergoing MRI scans, an interview was conducted with each participant by a trained radiology department technician. Following the interview, a checklist-based questionnaire was administered, which consisted of two sections: demographic information and research-specific characteristics. All data obtained from the participants was systematically recorded in a structured format.

## Statistical Analysis

The collected data was analyzed using SPSS software version 25 (IBM Corp. Released 2017). Parametric variables were analyzed using Chi-square tests and Fisher’s exact test when needed, with a significance level of 0.05.

## References

[1] Doberstein CA, Torabi R, Klinge PM. Current concepts in the pathogenesis, diagnosis, and management of type I Chiari malformations. RI Med J. 2017; 100(6): 47–9. https://www.ncbi.nlm.nih.gov/pubmed/28564670 28564670

[2] Kahn EN, Muraszko KM, Maher CO. Prevalence of Chiari I malformation and syringomyelia. Neurosurgery Clinics. 2015; 26(4): 501–7. DOI: 10.1016/j.nec.2015.06.006 26408058

[3] Milhorat TH, Nishikawa M, Kula RW, Dlugacz YD. Mechanisms of cerebellar tonsil herniation in patients with Chiari malformations as guide to clinical management. Acta Neurochirurgica. 2010; 152: 1117–27. DOI: 10.1007/s00701-010-0636-3 20440631 PMC2887504

[4] Dyste GN, Menezes AH, VanGilder JC. Symptomatic Chiari malformations: An analysis of presentation, management, and long-term outcome. Journal of Neurosurgery. 1989; 71(2): 159–68. DOI: 10.3171/jns.1989.71.2.0159 2746341

[5] Atkinson J, Kokmen E, Miller GM. Evidence of posterior fossa hypoplasia in the familial variant of adult Chiari I malformation: Case report. Neurosurgery. 1998; 42(2): 401–3; discussion 4. DOI: 10.1097/00006123-199802000-00129 9482195

[6] Paul KS, Lye RH, Strang FA, Dutton J. Arnold-Chiari malformation: Review of 71 cases. Journal of Neurosurgery. 1983; 58(2): 183–7. DOI: 10.3171/jns.1983.58.2.0183 6848674

[7] Elster AD, Chen M. Chiari I malformations: Clinical and radiologic reappraisal. Radiology. 1992; 183(2): 347–53. DOI: 10.1148/radiology.183.2.1561334 1561334

[8] Oakes W. Chiari malformation, hydromyelia, syringomyelia. Neurosurgery; 1985.

[9] Sathi S, Stieg PE. “Acquired” Chiari I malformation after multiple lumbar punctures: Case report. Neurosurgery. 1993; 32(2): 306–9. https://www.ncbi.nlm.nih.gov/pubmed/8437671. DOI: 10.1097/00006123-199302000-00023 8437671

[10] Buell TJ, Heiss JD, Oldfield EH. Pathogenesis and cerebrospinal fluid hydrodynamics of the Chiari I malformation. Neurosurgery Clinics. 2015; 26(4): 495–9. DOI: 10.1016/j.nec.2015.06.003 26408057 PMC5140100

[11] Huang PP, Constantini S. “Acquired” Chiari I malformation: Case report. Journal of Neurosurgery. 1994; 80(6): 1099–102. DOI: 10.3171/jns.1994.80.6.1099 8189267

[12] Pillay PK, Awad IA, Little JR, Hahn JF. Symptomatic Chiari malformation in adults: A new classification based on magnetic resonance imaging with clinical and prognostic significance. Neurosurgery. 1991; 28(5): 639–45. https://www.ncbi.nlm.nih.gov/pubmed/1876240. DOI: 10.1227/00006123-199105000-00001 1876240

[13] Sahuquillo J, Rubio E, Poca M-A, et al. Posterior fossa reconstruction: A surgical technique for the treatment of Chiari I malformation and Chiari I/syringomyelia complex--preliminary results and magnetic resonance imaging quantitative assessment of hindbrain migration. Neurosurgery. 1994; 35(5): 874–85. DOI: 10.1227/00006123-199411000-00011 7838336

[14] Sakamoto H, Nishikawa M, Hakuba A, et al. Expansive suboccipital cranioplasty for the treatment of syringomyelia associated with Chiari malformation. Acta Neurochirurgica. 1999; 141(9): 949–61. DOI: 10.1007/s007010050401 10526076

[15] Pascual J, Oterino A, Berciano J. Headache in type I Chiari malformation. Neurology. 1992; 42(8): 1519. DOI: 10.1212/wnl.42.8.1519 1641146

[16] Stovner LJ. Headache associated with the Chiari type I malformation. Headache: The Journal of Head and Face Pain. 1993; 33(4): 175–81. DOI: 10.1111/j.1526-4610.1993.hed33040175.x 8496055

[17] Haroun RI, Guarnieri M, Meadow JJ, Kraut M, Carson BS. Current opinions for the treatment of syringomyelia and chiari malformations: Survey of the Pediatric Section of the American Association of Neurological Surgeons. Pediatric Neurosurgery. 2000; 33(6): 311–7. DOI: 10.1159/000055977 11182642

[18] Heffez DS, Broderick J, Connor M, et al. Is there a relationship between the extent of tonsillar ectopia and the severity of the clinical Chiari syndrome? Acta Neurochirurgica. 2020; 162(7): 1531–8. DOI: 10.1007/s00701-019-04171-1 31873796

[19] Toldo I, Tangari M, Mardari R, et al. Headache in children with Chiari I malformation. Headache: The Journal of Head and Face Pain. 2014; 54(5): 899–908. DOI: 10.1111/head.12341 24766291

[20] Bezuidenhout AF, Chang Y-M, Heilman CB, Bhadelia RA. Headache in Chiari malformation. Neuroimaging Clinics. 2019; 29(2): 243–53. DOI: 10.1016/j.nic.2019.01.005 30926114

[21] Society HCSotIH. The international classification of headache disorders. Cephalalgia. 2004; 24(1): 9–160. 14979299 10.1111/j.1468-2982.2003.00824.x

[22] Riveira C, Pascual J. Is Chiari type I malformation a reason for chronic daily headache? Current Pain and Headache Reports. 2007; 11(1): 53–5. DOI: 10.1007/s11916-007-0022-x 17214922

[23] Furuya K, Sano K, Segawa H, Ide K, Yoneyama H. Symptomatic tonsillar ectopia. Journal of Neurology, Neurosurgery & Psychiatry. 1998; 64(2): 221–6. DOI: 10.1136/jnnp.64.2.221 9489535 PMC2169970

[24] Curone M, Valentini LG, Vetrano I, et al. Chiari malformation type 1-related headache: The importance of a multidisciplinary study. Neurological Sciences. 2017; 38(1): 91–3. DOI: 10.1007/s10072-017-2915-8 28527081

[25] Beretta E, Vetrano IG, Curone M, et al. Chiari malformation-related headache: Outcome after surgical treatment. Neurological Sciences. 2017; 38(1): 95–8. DOI: 10.1007/s10072-017-2950-5 28527074

